# The N-terminus and alpha-5, alpha-6 helices of the pro-apoptotic protein Bax, modulate functional interactions with the anti-apoptotic protein Bcl-x_L_

**DOI:** 10.1186/1471-2121-8-16

**Published:** 2007-05-23

**Authors:** Neha Parikh, Caroline Koshy, Vaigundan Dhayabaran, Lakshmi R Perumalsamy, R Sowdhamini, Apurva Sarin

**Affiliations:** 1 National Centre for Biological Sciences, Bellary Road, Bangalore 560065, Karnataka, India

## Abstract

**Background:**

Bcl-2 family proteins are key regulators of mitochondrial integrity and comprise both pro- and anti-apoptotic proteins. Bax a pro-apoptotic member localizes as monomers in the cytosol of healthy cells and accumulates as oligomers in mitochondria of apoptotic cells. The Bcl-2 homology-3 (BH3) domain regulates interactions within the family, but regions other than BH3 are also critical for Bax function. Thus, the N-terminus has been variously implicated in targeting to mitochondria, interactions with BH3-only proteins as well as conformational changes linked to Bax activation. The transmembrane (TM) domains (α5-α6 helices in the core and α9 helix in the C-terminus) in Bax are implicated in localization to mitochondria and triggering cytotoxicity. Here we have investigated N-terminus modulation of TM function in the context of regulation by the anti-apoptotic protein Bcl-x_L_.

**Results:**

Deletion of 29 amino acids in the Bax N-terminus (Bax 30–192) caused constitutive accumulation at mitochondria and triggered high levels of cytotoxicity, not inhibited by Bcl-x_L_. Removal of the TM domains (Bax 30–105) abrogated mitochondrial localization but resulted in Bcl-x_L _regulated activation of endogenous Bax and Bax-Bak dependent apoptosis. Inclusion of the α5-α6 helices/TMI domain (Bax 30–146) phenocopied Bax 30–192 as it restored mitochondrial localization, Bcl-x_L _independent cytotoxicity and was not dependent on endogenous Bax-Bak. Inhibition of function and localization by Bcl-x_L _was restored in Bax 1–146, which included the TM1 domain. Regardless of regulation by Bcl-x_L_, all N-terminal deleted constructs immunoprecipitated Bcl-x_L_and converged on caspase-9 dependent apoptosis consistent with mitochondrial involvement in the apoptotic cascade. Sub-optimal sequence alignments of Bax and Bcl-x_L _indicated a sequence similarity between the α5–α6 helices of Bax and Bcl-x_L_. Alanine substitutions of three residues (T14A-S15A-S16A) in the N-terminus (Bax-Ala3) attenuated regulation by the serine-threonine kinase Akt/PKB but not by Bcl-x_L _indicative of distinct regulatory mechanisms.

**Conclusion:**

Collectively, the analysis of Bax deletion constructs indicates that the N-terminus drives conformational changes facilitating inhibition of cytotoxicity by Bcl-x_L_. We speculate that the TM1 helices may serve as 'structural antagonists' for BH3-Bcl-x_L _interactions, with this function being regulated by the N-terminus in the intact protein.

## Background

Bcl-2 family proteins are central to the regulation of mitochondrial integrity and cell death pathways that converge on this organelle [1,2]. This family comprises both pro-survival (Bcl-2, Bcl-x_L_, Mcl-1, Bcl-w) and pro-apoptotic (Bax, Bak, Bim, Bid, Bad) proteins. All members of this family are characterized by the presence of BH (Bcl-2 homology) domain(s) [3,4]. Bax is a multidomain pro-apoptotic member of the Bcl-2 family, characterized by the presence of BH 1–3 domains. The BH3 domain regulates Bax homodimerization as well as heterodimerization with Bcl-2/Bcl-x_L_[5,6], such that the Bax-BH3 domain docks into the hydrophobic groove formed by BH1, BH2 and BH3 domains of the anti-apoptotic proteins [7-9]. Selectivity in interactions between pro- and anti-apoptotic members of the Bcl-2 family has been reported [10].

Changes in Bax conformation and localization are characteristic of many cells undergoing apoptosis [11-13]. In dying cells a conformationally distinct form of (activated) Bax accumulates at the mitochondrial outer membrane triggering the release of apoptogenic intermediates sequestered in the mitochondrial intermembrane space [14]. Several studies have yielded insights on the molecular mechanisms regulating Bax activation [15-18] and the N-terminus is implicated in this function. Controlled cleavage of the Bax N-terminus by calpain, a Ca^2+^-dependent cysteine protease generates a dominantly active form of Bax [19-21]. Short forms of Bax deleted of the first 28 or 32 amino acids are highly cytotoxic, largely associated with mitochondrial membrane and poorly inhibited by ectopically expressed Bcl-2 and Bcl-x_L _[22,23]. Two splice variants Bax ψ and Bax κ lack 19 amino acids present at the N-terminal of Bax-α and are potent inducers of cytotoxicity when ectopically expressed in cells [24,25].

Specific regions in the N and C terminus are implicated in the regulated mitochondrial targeting of Bax [26-28]. However, recombinant Bax protein lacking these regions retains mitochondrial targeting and insertion capabilities [27,29,30] because of two putative transmembrane (TM) regions, one encompassing the α5–α6 helices (amino acids 110–146) and the second the α9 helix (amino acids 172–188) at the C-terminus [31]. These are referred to as TM1 and TM2 respectively in this study. Recent experiments have shown that both TM1 and TM2 span the lipid bilayer in the membrane fraction of cells undergoing apoptosis [32]. Additionally other reports have identified a role for the α5–α6 helices in mitochondrial membrane insertion and cytochrome c release [29,33]. In the present study the TM1 domain and N-terminus have been characterized in the context of regulation of activity by Bcl-x_L_. Specifically, the experiments suggest that interactions between the N-terminus and TM1 region in intact Bax, modulate susceptibility to inhibition by Bcl-x_L_.

## Results

### Apoptosis triggered by a Bax N-terminal deletion mutant (Bax 30–192) is poorly inhibited by the anti apoptotic proteins, Bcl-2 and Bcl-x_L_

**Figure 1:**
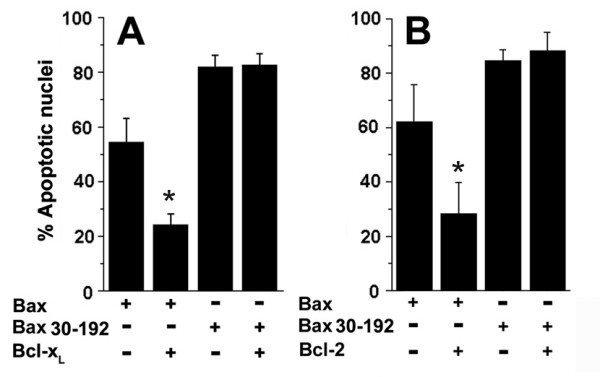
**Inhibition of Bax and Bax 30–192 by Bcl-2 and Bcl-x_L_**. A, GFP (1 μg), Bax-GFP (3 μg) or Bax 30–192-GFP (3 μg) with or without Bcl-x_L _(5 μg) were transfected in Jurkat cells. GFP positive cells were assessed for apoptotic nuclear damage. B, Jurkat cells were transfected with GFP (1 μg), Bax-GFP (3 μg) or Bax 30–192-GFP (3 μg) with or without Bcl-2 (5 μg) and assessed for apoptotic nuclear damage 12 hours post-transfection. The data are presented as mean ± SD derived from three-four independent experiments. *p < 0.001

Apoptosis triggered by a deletion mutant that lacks the first 29 amino acids (Bax 30–192) was poorly inhibited by Bcl-x_L _(Figure 1A) or Bcl-2 (Figure 1B) in the Jurkat T-cell line. Since Bcl-2 or Bcl-x_L _function may be modulated by other pro-apoptotic proteins present in mammalian cells, we turned to the heterologous system of *Drosophila melanogaster*, which expresses only one pro- and one anti-apoptotic protein of the Bcl-2 family [34]. We hypothesized that by expressing these proteins in  *Drosophila*, modulation by an endogenous modifier (if indeed it occurs)  will be independent of the construct being tested and will be equivalent  in all conditions.

**Figure 2:**
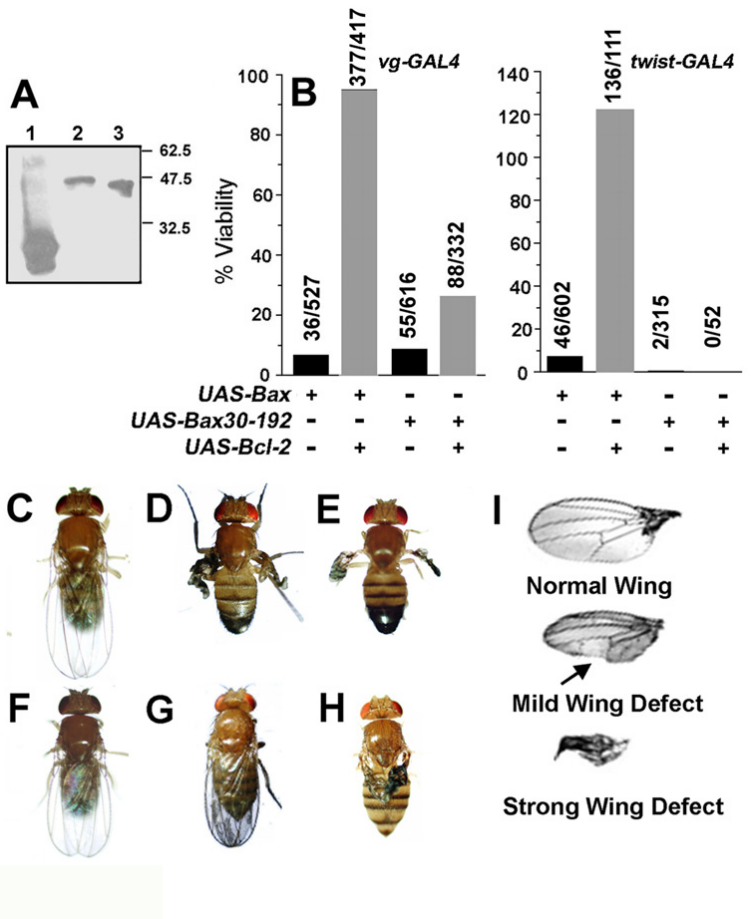
**The N-terminal deletion mutant is not regulated by Bcl-2 in *Drosophila melanogaster***. A, Cell lysates of S2 cells transfected with UAS-GFP (lane 1), UAS-Bax-GFP (lane 2) or UAS-Bax 30–192-GFP (lane 3) along with actin-GAL4 for 24 hours were assessed by western blot analysis using an anti -GFP antibody. B, Viability of flies was assessed in the indicated UAS transgenic lines driven at 25°C by vg-GAL4 (left panel) or twist-GAL4 (right panel). Viability is represented as the percentage of eclosed adult flies expressing Bax-GFP or Bax 30–192-GFP with respect to the appropriate sibling controls. The numbers over each bar indicate the total number of flies screened in the group. C-H, Wing phenotypes in vg-GAL4 flies sibling controls (C); transgenic fly lines of UAS-Bax-GFP (D); UAS-Bax 30–192-GFP (E); Bcl-2 (F); Bax-GFP + Bcl-2 (G) or Bax 30–192-GFP + Bcl-2 (H). I, Wing phenotypes of flies expressing Bax-GFP or Bax 30–192-GFP were categorized as illustrated. Representative images are shown. Arrow indicates notched margin of the wing obtained in few animals.

Bax-GFP, Bax 30–192-GFP and Bcl-2 were cloned into the pUAST vector and expression of the tagged proteins at the appropriate molecular weights confirmed in the *Drosophila *S2 cell line (Figure 2A). Transgenic fly lines of Bax, Bax 30–192 and Bcl-2 were generated by independent P-element transformation of the UAS-Bax-GFP (BA64III and BA13I), UAS-Bax30–192-GFP (DBA8II and DBA8IA) and UAS-Bcl-2 (BC57I and BC24III) constructs. Using the *UAS-GAL4 *system both Bax and Bax 30–192 were lethal to flies when driven in various tissues while Bcl-2 expression alone was not lethal (Table 1). This allowed us to test for the rescue of lethality by Bcl-2.

**Table 1:** Percent viability with tissue specific *GAL4 *drivers

	***vg- GAL4***	***twist- GAL4***	***scalloped- GAL4***	***eyeless- GAL4***	***collagen- GAL4***
***UAS-Bax-GFP***	6.8	7.6	0	0	0
(BA64III)	(36/527)	(46/602)	(0/141)	(0/96)	(0/130)
***UAS-Bax30–192-GFP***	8.9	0.6	1.6	0	0
(DBA8II)	(55/616)	(2/315)	(1/59)	(0/120)	(0/137)
***UAS-Bcl-2***	90.4	104	97.5		
(BC57I)	(377/417)	(259/248)	(39/40)		

*UAS *transgenic lines for Bax-GFP (BA64III) or Bax 30–192-GFP (DBA8II) or Bcl-2 (BC57I) were screened for lethality using various tissue specific *GAL4 *lines as indicated. Percent viability was calculated by assessing the number of Bax-GFP or Bax 30–192-GFP or Bcl-2 expressing flies compared to the appropriate sibling control that eclosed at 25°C. Numbers in parenthesis indicate animals screened for analysis.

For these experiments, *vg-GAL4 *and *twist-GAL4 *lines were used to drive expression largely in the dorsal margins of wings and mesoderm respectively. When driven along with Bax (BA64III, BC57I) using *vg-GAL4 *(Figure 2B*left panel*) or *twist-GAL4 *(Figure 2B, *right panel*) Bcl-2 rescued Bax induced lethality whereas Bax 30–192 (DBA8II, BC57I) induced lethality was only marginally rescued. Flies expressing Bax or Bax 30–192 (using *vg-GAL4*) present deformity in wings to various degrees (Figure 2I). All the flies that emerged for Bax (BA64III) and Bax 30–192 (DBA8II) presented a strong wing defect (Figure 2D and 2E). Bcl-2 co expression with Bax in the double transgenic fly line BA64III, BC57I resulted in complete rescue of the wing defect (Figure 2G) while there was no change in the severe wing defect in Bax 30–192 and Bcl-2 (DBA8II, BC57I) expressing flies (Figure 2H). Additional transgenic fly lines of *UAS-Bax *(BA13I), *UAS-Bax 30–192*(DBA8IA) and *UAS-Bcl-2 *(BC24III) were tested for viability and wing defect using *vg-GAL4 *with similar results (Table 2). Thus, the experiments using the heterologous model system of *Drosophila melanogaster *recapitulated the observation of inefficient inhibition of Bax 30–192 by anti-apoptotic proteins in mammalian cells.

**Table 2:** Percent viability and extent of wing defect with *vg-GAL*4 for additional *UAS *transgenic lines

	**% viability *vg-GAL4***	**% viable flies strong wing defect**	**% viable flies mild wing defect**	**% viable flies no wing defect**
***UAS-Bax-GFP***	9.5	41.3	55.1	0.03
(BA13I)	(29/303)	(12/29)	(16/29)	(1/29)
***UAS-Bax30–192-GFP***	12	65.2	30.4	4.3
(DBA8IA)	(26/216)	(15/26)	(7/26)	(1/26)
***UAS-Bax-GFP;***	78.6	0	0	100
***UAS-Bcl-2 ***(BA13I;BC24III)	(48/61)	(0/48)	(0/48)	(48/48)
***UAS-Bax30–192- GFP;***	35.09	84.9	15.09	0
***UAS-Bcl-2 ***(DBA8IA; BC24III)	(53/151)	(45/53)	(8/53)	(0/53)

Additional *UAS *transgenic lines for Bax-GFP (BA13I) or Bax 30–192-GFP (DBA8IA) or Bax-GFP+Bcl-2 (BA13I; BC24III) or Bax 30–192-GFP+Bcl-*2 *(DBA8IA; BC24III) driven by *vg*-*GAL4 *were assessed for the viability of flies at 25°C as described in Figure 2B. The eclosed adult flies expressing Bax-GFP or Bax 30–192-GFP were analyzed for the extent of wing defect and categorized as depicted in Figure 2I. The percentage of flies for the wing defect was calculated with respect to the total number of eclosed flies expressing Bax-GFP or Bax 30–192-GFP. Numbers in parenthesis indicate flies screened for analysis.

### Apoptosis triggered by transmembrane -1 (TM1) deletion mutants is inhibited by Bcl-x_L_

**Figure 3:**
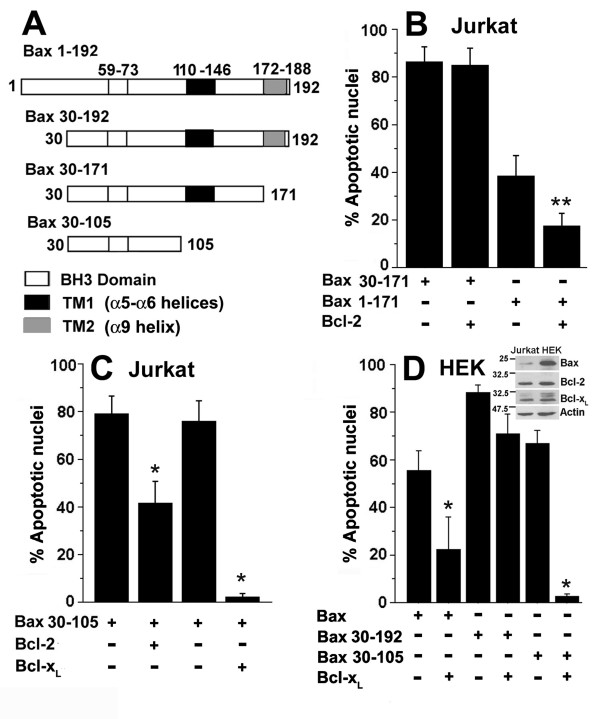
**Apoptosis triggered by TM deletion mutants is inhibited by Bcl-x_L_**. A, Schematic representation of the GFP tagged Bax constructs used in functional assays. The numerals indicate the amino acid residue number in the Bax protein (192 amino acids). TM1: transmembrane domain 1, TM2: transmembrane domain 2. B-D, Apoptotic nuclear damage in cells assessed 12–15 hours post-transfection in B, Jurkat cells transfected with GFP, Bax 30–171-GFP or Bax 1–171-GFP with or without Bcl-2 as indicated. The data in the figure is normalized to nuclear damage in GFP or GFP + Bcl-2 transfected cells. C, Jurkat cells transfected with GFP, Bax 30–105-GFP (3 μg) with Bcl-2 (3 μg) or Bcl-x_L _(3 μg). D, HEK cells transfected with Bax-GFP (1 μg), Bax 30–192-GFP (1 μg) or Bax 30–105-GFP (1 μg) with or without Bcl-x_L _(1 μg). Inset, shows the levels of Bax, Bcl-2 and Bcl-x_L _as determined by western blot analysis of the Jurkat and HEK cell lines. The data are presented as mean ± SD derived from a minimum of three independent experiments. **p < 0.01; *p < 0.001.

To assess the contributions of the TM domains in Bax 30–192 cytotoxicity, additional regions were deleted (Figure 3A). Apoptosis triggered by a mutant devoid of the C-terminal TM2 domain (Bax 30–171) is Bcl-2 independent, whereas apoptosis triggered by Bax 1–171 is inhibited (Figure 3B). However, it should be noted that apoptosis triggered by this construct was considerably lower than that triggered by full-length Bax thereby confounding interpretation of the data. Bax 30–105 which lacks any putative or experimentally proven TM domain but retains the BH3 domain triggered high levels of apoptotic damage, which was blocked by Bcl-x_L _or Bcl-2 (Figure 3C).

We then tested the deletion mutants for regulation by Bcl-x_L _in the HEK cell line as the larger size and adherent nature of the cells facilitates assessment of apoptotic damage and subcellular distribution of GFP-tagged proteins. Bax induced apoptosis was effectively blocked by Bcl-x_L_; apoptosis triggered by Bax 30–192 was poorly inhibited, and apoptosis triggered by Bax 30–105 was blocked by ectopically expressed Bcl-x_L _in this cell line (Figure 3D). Jurkat cells express lower levels of Bax protein compared to the HEK cell line arguing against interactions with endogenous Bax accounting for the responses of the deletion mutants.

### Bcl-x_L _regulation of Bax 30–192 and Bax 30–105 colocalization with Bid and Bim

**Figure 4:**
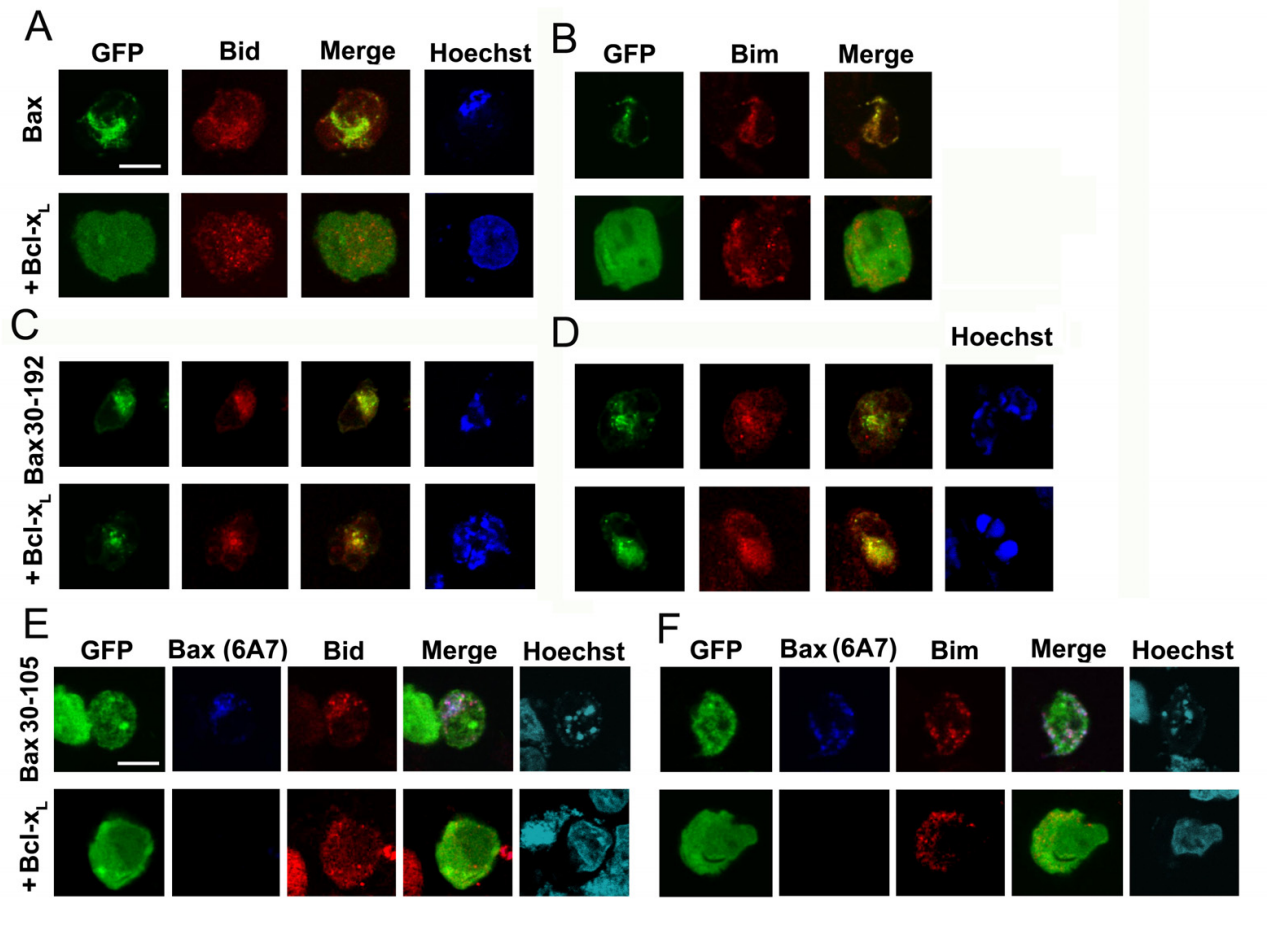
**Bcl-x_L _regulation of colocalization with Bid and Bim in HEK cells**. A and B, HEK cells transfected with Bax or Bax and Bcl-x_L _were stained for Bid or Bim. C and D, As in A, except that cells were transfected with Bax 30–192 with or without Bcl-x_L_. E and F, Cells transfected with Bax 30–105 with and without Bcl-x_L _were stained with clone 6A7 and Bid or Bim. Nuclear morphology was assessed by staining with Hoechst 33342. Single confocal planes of cells imaged at 60× magnification are shown. Scale bar: 10 microns.

The first alpha helix (amino acids 16–35) in the Bax N-terminus is implicated in interactions with activator BH3-only proteins [15,28,29]. In apoptotic cells, Bax clusters co-localized with Bid or Bim (Figure 4A and 4B, upper panel). In cells expressing Bcl-x_L_, Bid and Bim are evenly distributed and do not overlap Bax-GFP (Figure 4A and 4B, lower panel). Bax 30–192-GFP tends to coalesce into a large cluster and overlaps with regions of intense staining with Bid or Bim (Figure 4C and 4D upper panel). However, there is no change in the largely punctate distribution of Bax 30–192 or the overlap with Bid or Bim in cells co-expressing Bcl-x_L _(Figure 4C and 4D, lower panel).

**Figure 5:**
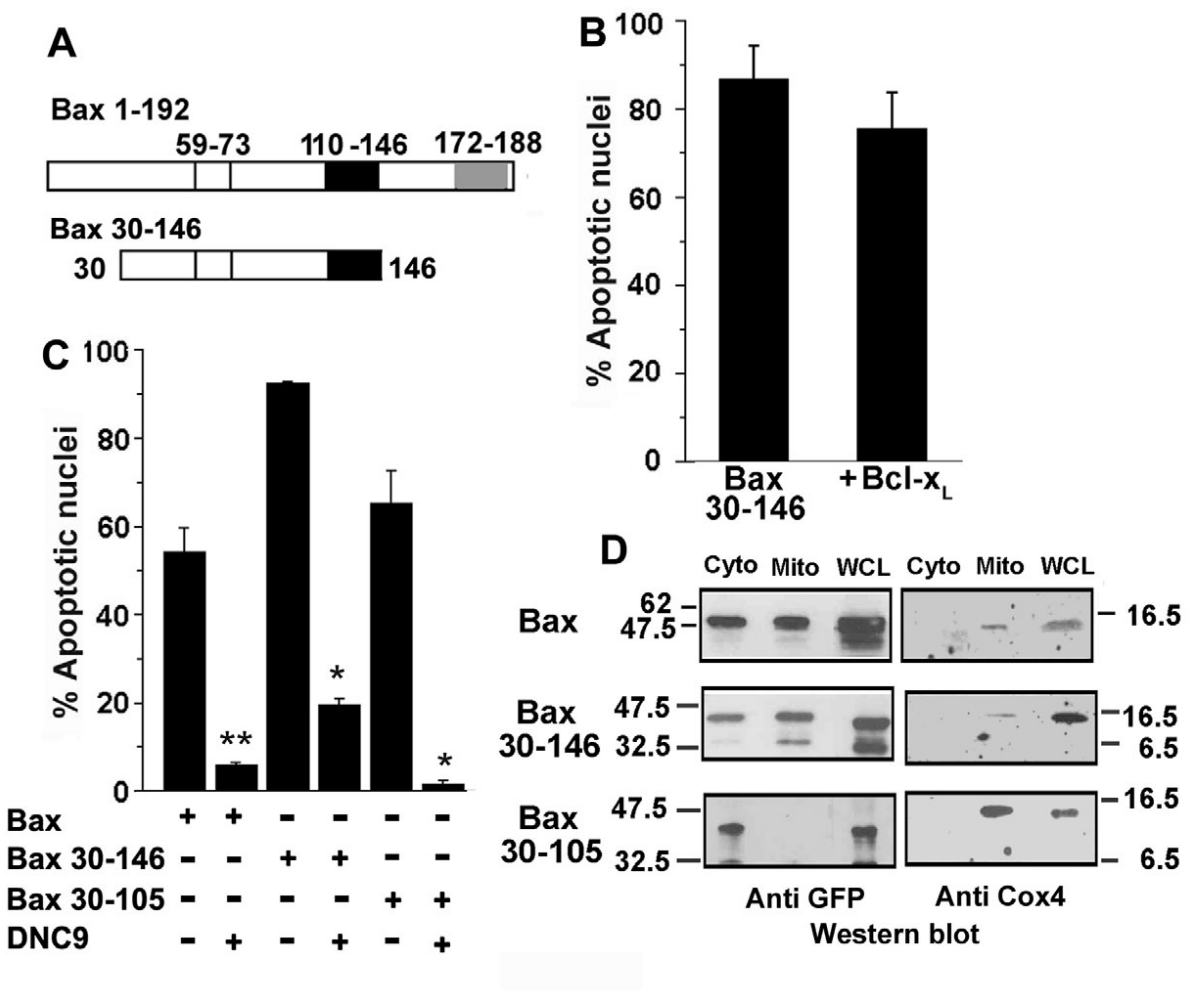
**The TM1 domain regulates inhibition by Bcl-x_L_**. A, Schematic representation of Bax and Bax 30–146. B, HEK cells transfected with Bax 30–146-GFP (1 μg) with or without Bcl-x_L _(1 μg) and assessed for apoptotic nuclear damage after 15 hours of culture. Data are normalized for apoptotic damage triggered by GFP alone in both conditions. C, HEK cells were transfected with 1 μg GFP or Bax-GFP or Bax 30–146-GFP or Bax 30–105-GFP with or without 2 μg dominant negative caspase 9 (DNC9). Apoptotic nuclear damage was assessed after 15 hours of culture. Data is normalized to damage in cells transfected with GFP alone or GFP + DNC9 transfected cells. D, HEK cells transfected with Bax-GFP or Bax 30–146-GFP or Bax 30–105-GFP were harvested after 15 hours of culture and subjected to sub-cellular fractionation. The cytosolic (Cyto) and membrane/mitochondrial (Mito) fractions were assessed for GFP and Cox4 distribution by western blot analysis. WCL: whole cell lysate. Values in the anti-GFP blots are derived from densitometry analysis and indicate the amount of GFP detected in the specific fraction relative to the total signal in the mitochondria and cytosol fractions. The data are presented as mean ± SD derived from a minimum of three independent experiments. **p < 0.01; *p < 0.001.

Since Bax 30–105 triggered Bcl-x_L _dependent apoptosis although it did not localize to mitochondria (Figure 5D), we asked if it triggered the activation of endogenous Bax. Changes in Bax conformation consistent with its activation reveal an epitope in the N-terminus that can be detected using a specific antibody clone 6A7 [35]. Bax 30–105 lacks the relevant section of the N-terminus, and 6A7 reactivity in cells expressing Bax 30–105 indicated the activation of endogenous Bax (Figure 4E). This reactivity was lost in cells that express Bax 30–105 and Bcl-x_L _(Figure 4F). Further, endogenous Bax as detected by the reactivity of clone 6A7 colocalized with Bid and Bim and this was regulated by Bcl-x_L _(Figure 4E and 4F).

### In the absence of the N-terminus, the TM1 domain attenuates inhibition by Bcl-x_L _but triggers caspase-9 dependent apoptosis

A construct that included the TM1 domain comprising the α5–α6 helices, but not the N-terminal 29 amino acids (Bax 30–146) (Figure 5A) triggered Bcl-x_L _independent cytotoxicity (Figure 5B) indicating that the TM1 domain interfered with the anti-apoptotic function of Bcl-x_L_. Since Bax function converges on the activation of caspase-9 [36] we analyzed apoptotic damage induced by Bax, Bax 30–146 or Bax 30–105 when co-expressed with a construct that is a dominant-negative inhibitor of caspase-9 (DNC9) [37,38]. Apoptosis triggered by all the mutants tested, including Bax 30–192 (data not shown) was blocked by the co-transfection of DNC9 in HEK cells (Figure 5C) and in the Jurkat cell line (data not shown). The data show that all the constructs tested activate a caspase-9 mediated apoptotic cascade as has been established for Bax.

Sub-cellular fractionation of HEK cells (Figure 5D) transfected with the relevant constructs showed that Bax is present in both the cytosolic and mitochondrial fractions, whereas increased amounts of Bax 30–146 was detected in the mitochondrial fractions (Figure 5D, *upper *and *middle panel*). In many experiments, the distribution of Bax 30–146 and Bax 30–192 was skewed to the mitochondrial fraction (not shown). Expectedly, Bax 30–105 was consistently detected only in the cytosolic fraction. The mitochondrial matrix protein Cox-4 was used to ascertain purity of the fractions.

### Bcl-x_L _associates with but does not regulate the localization of N-terminal deletion constructs

**Figure 6:**
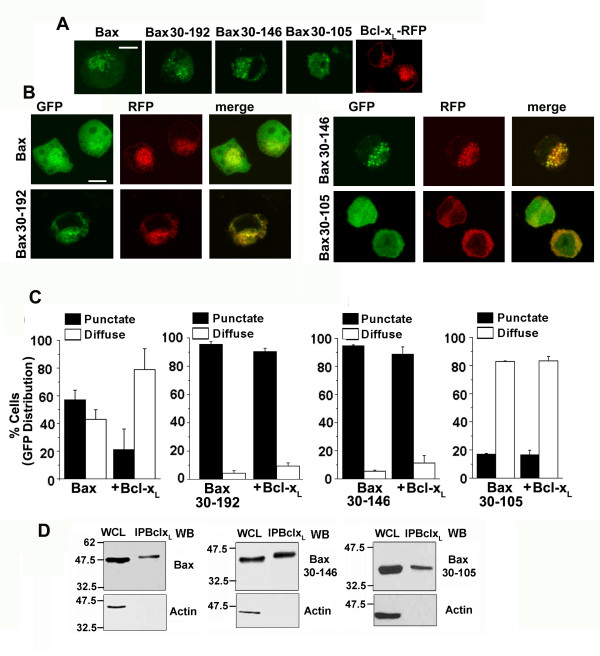
**The Bax N-terminus or TM1 domain is not essential for association with Bcl-x_L_**. A, HEK cells transfected with Bax-GFP, Bax 30–192GFP, Bax 30–146 GFP, Bax 30–105 GFP or RFP-Bcl-x_L _were harvested after 15 hours and imaged using confocal microscopy. Representative images (processed as described in methods) are shown. Scale bar: 10 microns B, HEK cells transfected with each of the constructs in A and RFP-Bcl-x_L _were harvested 15 hours post-transfection. Confocal images for distribution of Bax constructs (green) and RFP-Bcl-xL (red) were assessed for co-localization (Merge, right panel). Scale Bar: 10 microns. Representative images (each corresponding to a single image field) are shown. C, From the experiment in B, cells positive for GFP and RFP were scored for punctate or diffuse patterns of GFP by microscopy. The data are representative of two independent analyses. D, HEK cells transfected with Bax-GFP + Bcl-x_L _(left panel) or Bax 30–146-GFP + Bcl-x_L _(middle panel) or Bax 30–105-GFP + Bcl-x_L _(right panel) were harvested after 15 hours of culture. Bcl-x_L _was immunoprecipitated from cell lysates and the immunoprecipitates assessed by western blot analysis for Bax or the mutant constructs using an antibody to GFP and for the presence of actin. WCL represents the CHAPS buffer solubilized whole cell lysate, which was used for immunoprecipitation.

Subsequently we assessed the cellular distribution of the constructs using GFP to report on Bcl-x_L _regulation of Bax localization. In the absence of exogenous Bcl-x_L_, the distribution of Bax, Bax 30–146 and Bax 30–192 is punctate in apoptotic cells whereas, Bax 30–105 is diffuse (Figure 6A). In cells co-transfected with Bcl-x_L _(RFP-Bcl-x_L_), Bax distribution is rendered diffuse (Figure 6B) as opposed to Bax 30–146 or Bax 30–192, where the punctate distribution is largely unchanged by Bcl-x_L _(Figure 6B). The distribution of Bax 30–105 was not changed by Bcl-x_L _(Figure 6B). The distribution of Bax in punctate or diffuse patterns in the presence or absence of Bcl-x_L _is plotted in Figure 6C. The values are derived from an assessment of approximately 200 cells in random fields in each experiment. This experiment shows that in Bax constructs which lack the first 29 amino acids Bcl-x_L _regulation of cellular distribution is compromised.

Ectopically expressed Bcl-x_L _immunoprecipitated cotransfected Bax, Bax 30–146 or Bax 30–105 (Figure 6D) indicating that association with Bcl-x_L_is not impaired in the absence of the first twenty nine residues in Bax protein.

### The Bax N-terminus regulates inhibition by Bcl-x_L_

**Figure 7:**
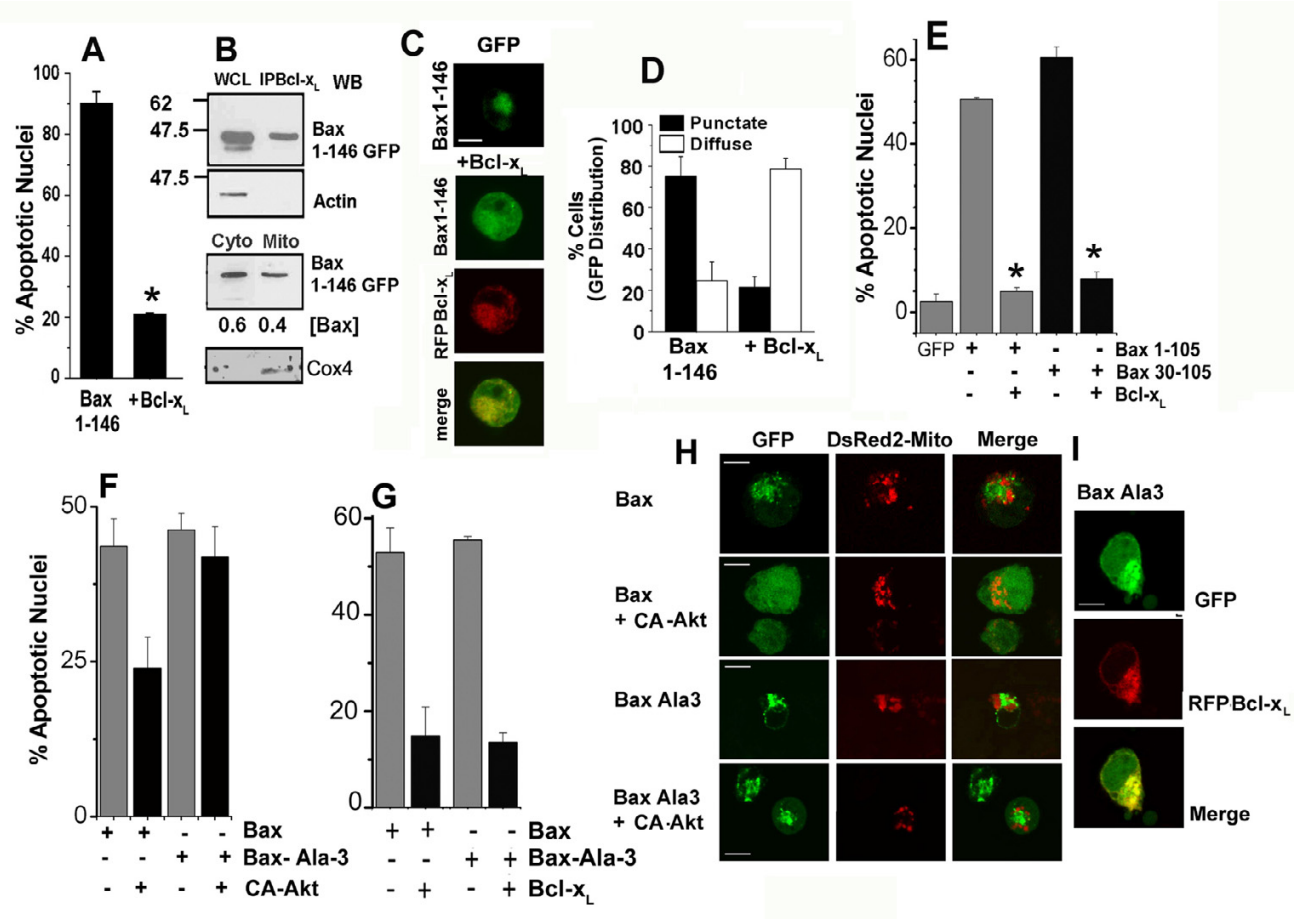
**The Bax N-terminus regulates susceptibility to inhibition by anti-apoptotic molecules**. A, Apoptotic nuclear damage (15 hours post-transfection) was assessed in HEK cells transfected with Bax 1–146-GFP with or without RFP-Bcl-x_L_. B, HEK cells transfected with Bax 1–146 GFP and Bcl-x_L _were harvested after 15 hours of culture and Bcl-x_L _immunoprecipitated from the cell lysate. The precipitated complex was analyzed for Bax 1–146-GFP and actin by western blot analysis. WCL is the CHAPS buffer-solubilized whole cell lysate used in the immunoprecipitation. HEK cells transfected with Bax 1–146-GFP were harvested 15 hours post-transfection and processed to enrich for the cytosol (Cyto) and the membrane/mitochondrial (Mito) fractions. The distribution of GFP and Cox4 was determined by western blot analysis. WCL represents the whole cell lysate. Numbers below the blots are the densitometric analysis of the GFP signal in the specific fraction relative to the total in the mitochondria and cytosol fractions. C, HEK cells expressing Bax 1–146 alone or Bax 1–146+RFP-Bclx_L _were assessed for distribution as described in 6B. E, Apoptotic nuclear damage was assessed in HEK cells 18–20 hours following transfection with combinations of constructs shown in the panels. F and G, Regulation of Bax and Bax-Ala3 induced apoptosis by constitutively active (CA)- Akt (F) and Bcl-x_L _(G). The data in all panels are presented as mean ± SD and are derived from three-five independent experiments. H and I, HEK cells transfected with Bax-GFP or Bax-Ala-3 with CA-Akt and Mito-dsRed2 or RFP-Bcl-x_L _where indicated were harvested 15 hours post-transfection. Confocal images for distribution of GFP tagged constructs of Bax (green) and Mito-dsRed2 (red) or RFP-Bcl-x_L _(red) were assessed for co-localization (Merge). Scale Bar: 10 microns. Representative images (each corresponding to a single image field) are shown. *p < 0.001

Since Bcl-x_L _poorly regulated Bax 30–146 or 30–192 despite the association with the protein, we tested if the N-terminus can modulate this function. Apoptosis triggered by Bax 1–146 which includes the N-terminus and the TM1 domain was efficiently blocked by RFP-Bcl-x_L _(Figure 7A). Bax 1–146 immunoprecipitates Bcl-x_L _and is detected in both mitochondrial and cytosolic fractions (Figure 7B). Furthermore, the punctate distribution of Bax 1–146 in apoptotic cells redistributes to a diffuse pattern in cells that co-express Bcl-x_L _(Figure 7C). The distribution of Bax 1–146 overlaps significantly with that of Bcl-x_L _(Figure 7C). The change in distribution of Bax 1–146 in response to co-expressed Bcl-x_L _is shown (Figure 7D). In a construct that lacks the TM domains, the N-terminus did not modulate Bax-BH3 function (Figure 7E).

Phosphorylation dependent modifications that change protein conformation regulate the apoptotic function of Bcl-2 family proteins. Bax-induced apoptosis is inhibited by the serine-threonine kinase Akt (Figure 7F and 7H). This regulation was compromised in a Bax construct (Bax-Ala3) with alanine substitutions of the Ser/Thr residues (T14, S15, S16) in the N-terminus which was refractory to inhibition by Akt (Figure 7F and 7H). The modification in the N-terminus however, did not interfere with Bcl-x_L _inhibition of Bax-Ala3 induced apoptosis (Figure 7G and 7I).

### The N-terminus regulates sensitivity to inductive stimuli and inhibition by Bcl-x_L_

**Figure 8:**
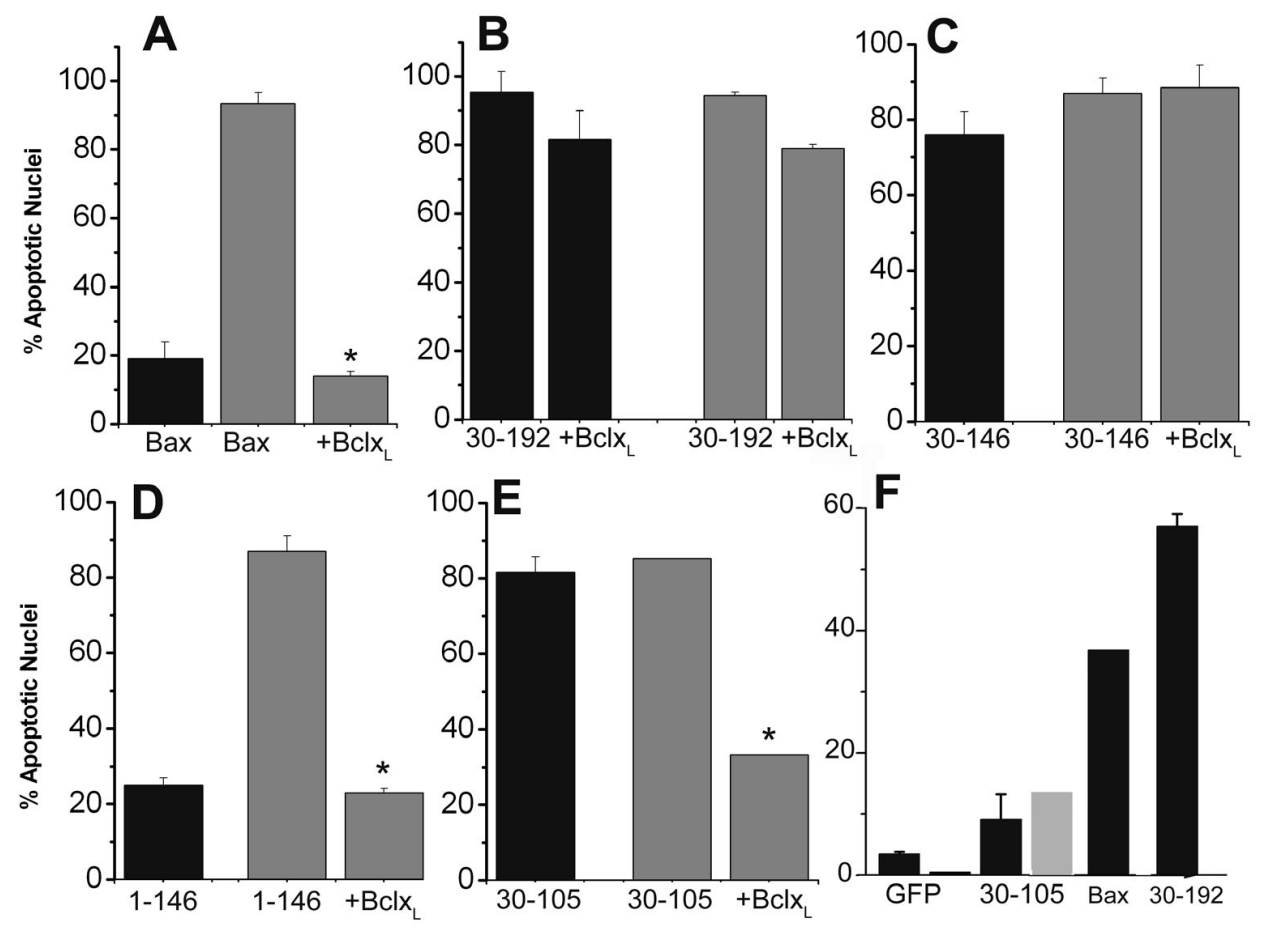
**Responses of Bax deletion mutants to an inductive stimulus**. A-E, Cos-7 cells were transfected with indicated constructs as described in methods and apoptotic nuclear damage assessed 3–4 hours after the addition of STS (gray bars). The bars in black indicate apoptotic damage in the absence of STS. F, MEF that are deficient for both Bax and Bak were assessed for susceptibility to Bax, Bax 30–105 and Bax 30–192. Bax 30–105 was tested in the presence (gray bars) or absence (black bars) of STS. *p < 0.001

The deletion mutants were tested in a cellular system where Bax activity is regulated by externally applied stimuli [11,39]. In Cos-7 cells, staurosporine (STS) triggered the translocation of ectopically expressed Bax and was a potent inducer of Bax-mediated cytotoxicity, which was inhibited in cells co-expressing Bcl-x_L _(Figure 8A). Bax 30–192 and Bax 30–146 localized into puncta (data not shown) or large clusters and triggered high levels of apoptotic damage which were not further enhanced by STS (Figure 8B and 8C) or inhibited by Bcl-x_L _(Figure 8B and 8C). Bax 1–146 is largely diffuse but also localizes as puncta and is a potent inducer of Bcl-x_L _dependent cytotoxicity in response to STS (Figure 8D). Bax 30–105 triggered apoptosis in the absence of STS although Bcl-x_L _inhibited apoptotic damage in the presence or absence of the apoptotic stimulus (Figure 8E).

The data thus far suggested that apoptosis triggered by Bax 30–146 and Bax 30–192 should not depend on endogenous Bax or Bak, whereas Bax 30–105 induced apoptosis may depend on these proteins. MEF deficient for both Bax and Bak were therefore tested for susceptibility to apoptosis by the Bax deletion mutant constructs. Confirming these predictions Bax and Bax 30–192 but not Bax 30–105 triggered apoptosis in Bax/Bak deficient cells (Figure 8F).

### TM1 helices of full-length Bax are structural antagonists to Bcl-x_L_

**Figure 9:**
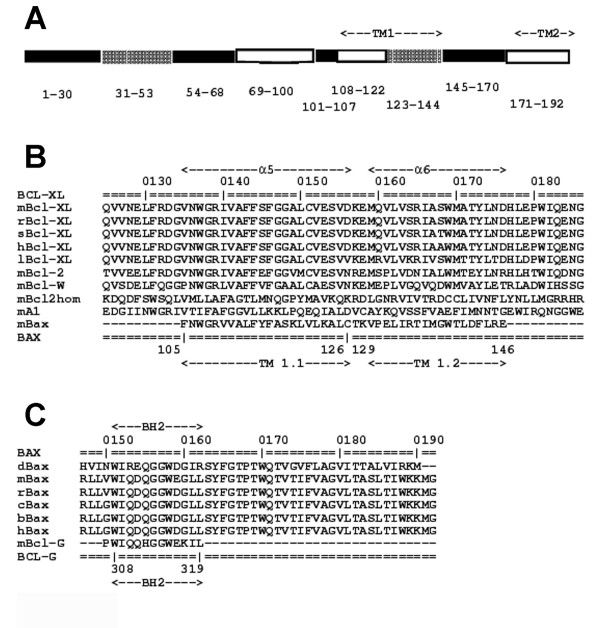
**Structural and sequence analyses of Bax and Bcl-x**_L_**proteins**. A, Schematic representation of the spatial interactions between different segments of full-length Bax. The homology model of Bax was examined using the program DIAL [40], originally meant for recognizing compact structural domains in proteins. Structural segments with similar shading form spatial clusters and subdomains. The BH2 region (residues 145–170) forms a cluster with the N-terminal 29-residues indicating spatial proximity between the two segments. B, Sub-optimal sequence alignment between the helices in TM1 region of representative pro-apoptotic family members and alpha-5 of anti-apoptotic proteins. Protein names and residue numbers are marked for the sake of clarity. C, As for B, but showing the alignment between the BH2 helix of pro-apoptotic proteins and Bcl-x_L_.

In an attempt to elucidate structural correlates if any, of the phenotypes presented by the mutants we turned to a sequence-based analysis of the interacting proteins. The three-dimensional structures of full-length Bax sequence and deletion mutants have been obtained from homology modeling, using MODELLER (version 8v1) starting from the human protein that has 92% sequence identity. The homology model of Bax was examined using the program DIAL [40]. The deletion experiments, using mutants lacking the first 29 residues, suggest that major structural alterations in full-length Bax involving the N-terminal 29 residues, influence the penultimate helix connecting TM1 and TM2 regions in the three-dimensional model of full-length Bax (Figure 9A). In order to test if there is structural equivalence between the anti-apoptotic protein Bcl-x_L _and regions in Bax, we examined full-length Bcl-x_L _and Bax for sub-optimal sequence similarity. A high gap penalty of 400 was employed to align the two sets of sequences using MALIGN [41]. In particular, we notice that the first helix of TM1 (TM1.1 helix) and the second helix of TM1 (TM1.2 helix) have significant sequence similarity with α-5 and α-6 of Bcl-x_L _and related anti-apoptotic proteins, respectively (Figure 9B). This is supported by previous structural studies where Bcl-x_L _was co-crystallized with BH3-region of Bim and both helices α-5 and α-6 of Bcl-x_L _in this complex interact with the BH3 of Bim [9]. This suggests that the presence of TM1 region could act as structural antagonists for the interaction of Bcl-x_L _with Bax. Furthermore, the BH2 region of Bcl-x_L _shows local sequence similarity with the penultimate BH2 helix in Bax (Figure 9C).

## Discussion

The BH3 domain is critical to several interactions within the Bcl-2 family [5-9] and the anti-apoptotic proteins Bcl-2 and Bcl-x_L _regulate Bax via interactions involving this domain [5,42]. However, mutations in the Bax-BH3 domain which disrupt interactions with pro-survival proteins do not always disrupt regulation by the latter [17,43-45]. The N-terminus of Bax is implicated in the regulation of its function by Bcl-2/Bcl-x_L _and activator BH3-only proteins [[15,22,23] and [46]]. Here we show that the BH3 and TM1 domains are adequate for association with Bcl-x_L _and localization to mitochondria respectively, but an intact N-terminus plays a non-redundant role in the regulation of Bax cytotoxicity by Bcl-x_L_.

In contrast to Bax [11,15,26], the distributions of Bax 30–192 or Bax 30–146 were largely unchanged by Bcl-x_L _(Figure 6B–C). The TM1 domain/α5–α6 helices (amino acids 110–146) in Bax bring about membrane insertion and cytochrome c release from mitochondria in cell-free assays [29,33]. The substitution of charged residues in the α5–α6 helices of Bax resulted in its constitutive localization to mitochondria, akin to gain-of-function mutants and increased binding to Bcl-x_L _[47]. Despite consistent differences from Bax in regulation by Bcl-x_L_, the N-terminal deletion mutants immunoprecipitated ectopically expressed Bcl-x_L _(Figure 6D). This is not unexpected as the association of apoptotic with anti-apoptotic proteins does not culminate in the inhibition of pro-apoptotic activity [17,43-45,48]. Further, Bax 30–192 triggered caspase-9 dependent apoptosis and localized with Bid and Bim conventionally associated with regulated activity of Bax [15,48,49] a distribution largely unchanged by Bcl-x_L_. In our experiments the effect of the TM1 domain was mitigated by the inclusion of the N-terminal region i.e. Bax-1–146 (Figure 7).

The anti-apoptotic proteins Bcl-2 and Bcl-x_L _are not functionally equivalent and the differences in the regulation of Bax 30–105 (Figure 3C) likely reflect differences in affinities of the mutant for the anti-apoptotic proteins. Such differences in functional interactions between pro-survival members and the pro-apoptotic or BH3 only proteins (BOPs) have been previously reported [17,50]. The BOPs are thought to be critical mediators of the activation of Bax/Bak although the mechanism underlying this activation is unresolved. Thus, 'activator" BOPs (Bid, Bim and Puma) have been reported to directly bind Bax and Bak triggering activation of the latter and are assisted in this function by "derepressor" BOP (Noxa, Bad etc.) which bind anti-apoptotic proteins [17]. On the other hand there is compelling evidence that BOP trigger the activation of Bax principally by binding Bcl-2/Mcl-1/Bcl-x_L _[48] obviating a requirement for direct interactions with Bax/Bak except in some conditions [51]. Bax 30–105 fulfills several criteria associated with BH3 domain only proteins triggering activation of endogenous Bax (which colocalized with Bid and Bim); Bcl-x_L _dependence and in the requirement for endogenous Bax-Bak for apoptotic function (Figure 3C–D, 4E–F and 8F).

We do not know the mechanism underlying the lack of regulation of Bax 30–192 and Bax 30–146 by Bcl-x_L_. The constitutive association of Bax 30–192 and 30–146 with Bcl-x_L _may arise from structural changes in the deletion mutants culminating in a greater affinity (compared to Bax) for the anti-apoptotic protein. This may permit the mutants to sequester ectopically expressed Bcl-x_L_. On the other hand the association may impose conformational changes on Bcl-x_L _such that the latter is rendered functionally inactive and thereby unable to regulate apoptotic damage. That the pattern of Bid distribution in cells expressing Bax or Bax 30–105 changes on co-expressing Bcl-x_L _but not in cells that express Bax 30–192 provides some support for this possibility.

**Figure 10:**
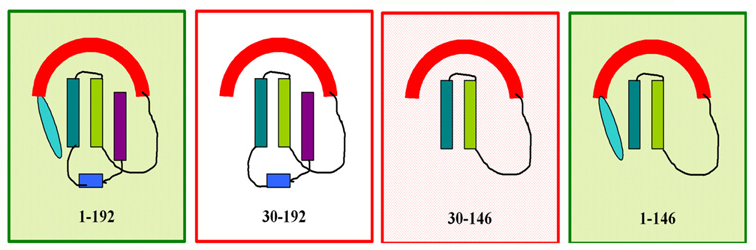
**A model summarizing the behavior of deletion mutants of Bax**. The deletion constructs are named according to the beginning and end residue numbers. Different regions of Bax: N-terminal 29 residues, BH3 helix, the first and second helices of TM1 region (TM1.1 and TM1.2), BH2 helix and TM2 region, are color-coded as cyan, red, green, blue and purple, respectively. Constructs whose apoptotic activity is regulated by Bcl-x_L _are in shaded boxes. The open boxes show deletion mutants not regulated by Bcl-x_L_.

Analysis of local sequence similarities indicate that sub-optimal alignments reside at the interacting site of Bcl-x_L _and Bax TM1 helices. Sub-optimal sequence alignment between TM1 of Bax and α5–α6 of Bcl-x_L _suggests that the TM1 might block a productive functional interaction with Bcl-x_L_. The absence of N-terminal residues in Bax leads to a dramatic effect in its regulation by Bcl-x_L _in apoptotic function. The deletion analysis of Bax suggests that the N-terminal residues that are spatially interacting with the BH2 helix are absolutely required for (and may drive) the normal and necessary structural alterations in TM1 helices such that the anti-apoptotic elements can regulate its function. These are currently being investigated by detailed molecular dynamics simulations as well as studying the effect of additional mutations in Bax. The TM2 region in Bax may not be a crucial structural antagonist to Bcl-x_L _since the removal of TM2 results in a mutant (Bax 30–171) whose apoptotic activity is not regulated by Bcl-x_L _in the Jurkat and the HEK cell lines. Thus we propose (as depicted in the model in Figure 10) that the N-terminal "switch" of 29 residues is necessary to produce the required structural alterations in Bax such that TM1.1 is displaced from the Bcl-x_L _interacting site. In the absence of the N-terminal switch, this natural "trigger" mechanism is abolished and the TM helices continue to act as 'structural valves' where the protein remains closed to inhibition by Bcl-x_L_-like proteins.

## Conclusion

Our experiments extend previous observations that the Bax N-terminus is an essential component in the regulation of Bax function by anti-apoptotic proteins such as Bcl-x_L_. Furthermore we propose that an intramolecular interaction between the N-terminus and the TM1 may be the mechanism underlying this regulation.

## Methods

### Cells and reagents

Jurkat, HEK and COS-7 cell lines and the Bax-Bak deficient MEF were maintained in culture in medium supplemented with antibiotics, glutamine (Invitrogen-Gibco, Carlsbad, CA) and 5 or 10% fetal calf serum (Hyclone, Logan, UT). Antibodies were procured from the following sources: to GFP and Bim from BD Biosciences (Franklin Lakes, NJ); to Bax and Bcl-2 from Santa Cruz (Santa Cruz, CA); to Cox-4 and and Alexa Fluor 555/Cy5 conjugated secondary antibodies from Invitrogen-Molecular Probes; to Bid from Cell Signaling Technology; to Bcl-x_L _from Abcam (Cambridge, UK); actin and clone 6A7 from Neomarker (Fremont, CA) respectively. Protein G agarose beads were from Pierce Biotechnology (Rockford, IL). Mouse or Rabbit True-Blot HRP (Ebiosciences, San Diego, CA) was used in the western blot analysis of immunoprecipitated proteins. All other chemicals were obtained from Calbiochem (San Diego, CA) or Invitrogen.

### Plasmids

A mammalian expression vector encoding murine full-length Bax was the kind gift of Peter Vandenabeele (University of Gent, Belgium). Bax-GFP and the deletion mutants were generated by PCR amplification and subcloning into the pEGFP-N1 vector (BD Clontech, Mountain View, CA) with GFP in-frame at the C- terminus. The following primers were used to generate the Bax and Bax mutants: Bax/Bax 1–171/Bax 1–146 forward 5' CCG CTC GAG ATG GAC GGG TCC GGG 3' Bax 30–192/Bax 30–171/Bax 30–146/Bax 30–105 forward 5' CCG CTC GAG ATG TTC ATC CAG GAT 3' Bax/Bax 30–192 reverse 5' CCC AAG CTT GCC CAT CTT CTT CCA 3' Bax 30–171/Bax 1–171 reverse 5' CCC AAG CTT CTG CCA TGT GGG 3' Bax 30–146/Bax 1–146 reverse 5'CCC AAG CTT CTC ACG GAG GAA 3' Bax 30–105 reverse 5' CCC AAG CTT GAA GTT GCC ATC 3'. The murine Bcl-2 plasmid was obtained from Upstate (Charlottesville, VA). The dominant negative caspase 9 construct was the kind gift of C. Vincenz (University of Michigan Medical School, Ann Arbor, MI) and the human Bcl-x_L _plasmid was from Richard J. Youle (National Institutes of Health, Bethesda, MD). CA-Akt was from Upstate (Charlottesville, VA). Monomeric RFP (mRFP) plasmid was originally from Roger Y. Tsien (University of California, San Diego, CA) and obtained through Satyajit Mayor (National Centre for Biological Sciences, Bangalore, India). Bcl-x_L _was subcloned into the pUSEAmp mammalian expression vector and was also subcloned at the 3' end of mRFP under the CMV promoter. Mito-DsRed was obtained from V. Sriram (National Centre for Biological Sciences, Bangalore, India). The sequence of all constructs was verified by automated sequencing (Microsynth, Balgach, Switzerland). The alanine substitution mutant Bax-Ala3 (substituted on T14, S15 and S16) was generated at Everogen (Moscow, Russia).

### Transient transfections and assay for nuclear damage

Jurkat and 2B4 cells were transfected by electroporation. Briefly 3–5 million cells were electroporated at 250 V and 960 μF and cultured for 12 hours prior to analysis. HEK and COS-7 cells were plated at 0.4–0.5 million cells/ml and MEF at 0.3 million/ml 12–15 hours prior to transfection. Cells were transfected using lipofectaime 2000 (Invitrogen, Carlsbad, CA) according to the manufacturer's instructions. Cos-7 cells were transfected with 0.2 μg of Bax constructs and 2 μg Bcl-x_L_. 12–15 hours later staurosporine was added at a final concentration of 1 μM and the cultures continued for an additional 3–4 hours before analysis.

Transfected cells were harvested and stained for 3 minutes at ambient temperatures in the dark using the Hoechst 33342 (1 μg/ml) and assessed for apoptotic nuclear damage. GFP positive cells were viewed under the UV filter of the fluorescence microscope and nuclear morphology scored for a minimum of 200 cells in each experimental condition. In a minimum of two experiments in each set of data samples were scored double-blind. Statistical significance of the differences between groups was assessed by the Student's t-test and is indicated by an * in the figures.

### Sub-cellular fractionation

The protocol followed has been previously described [52]. Briefly, 1 million cells were incubated in 100 μl homogenization buffer (250 mM sucrose, 20 mM Hepes, 10 mM KCl, 1.5 mM MgCl2, 1 mM EDTA, 1 mM EGTA, pH 7.2 supplemented with 2 μg/ml aprotinin, leupeptin and pepstatin) for 15 min on ice. Cells were mechanically disrupted by passing through a 26-gauge needle 15 times followed by a 30-gauge needle 15 times. Cell lysates were centrifuged at 750 g for 10 min at 4°C to remove unlysed cells and nuclei. The supernatant was centrifuged at 12,000 g for 20 min at 4°C to obtain the pellet enriched in mitochondria and the supernatant enriched for cytosolic proteins. Equivalent volumes of cytosolic and mitochondrial fractions were used for western blot analysis to assess the relative distribution of Bax and the mutants. The densitometry analysis of western blots was performed using Image Gauge software Version-3 (Fujifilm Science, Japan)

### Immunoprecipitation

In each group two million cells were harvested and lysed in 500 μl of CHAPS lysis buffer (1% CHAPS in 50 mM Tris-Cl, 150 mM NaCl, 1 mM EDTA, pH7.4 supplemented with 2 μg/ml aprotinin, leupeptin, pepstatin, 1 mM PMSF, 1 mM NaF and 1 mM Na_3_OV_4_) by 3 freeze thaw cycles in liquid nitrogen and 37°C followed by an incubation (30 minutes) at 4°C on a cell rotator. The lysate was centrifuged at 3000 rpm to remove debris and unlysed cells. The supernatant was incubated with 10 μg of antibody to Bcl-x_L_at 4°C for 1 hour on a cell mixer. 80 μl of washed Protein-G agarose beads were then added to the sample and mixed by gentle rotation for another 2 hours at 4°C. Beads were pelleted by centrifugation at 1700 rpm and subsequently washed 4 times with chilled PBS. After the final wash the beads-complex was pelleted by centrifugation at 2500 rpm and boiled in SDS lysis buffer for 10 minutes before western blot analysis.

### Immunochemistry and confocal imaging

Cells washed with PBS and fixed in 4% paraformaldehyde for 15 minutes at ambient temperature were permeabilized with 0.2% CHAPS in PBS (permeabilization buffer) for 30 minutes on ice and blocked for 1 hour on ice with 5% BSA diluted in permeabilization buffer. Cells were incubated with primary antibodies diluted in blocking buffer for 1–2 hours on ice followed by a wash with permeabilization buffer. The cells were finally incubated with Alexa Fluor 555 and/or Cy5 conjugated secondary antibodies diluted in blocking buffer for 1 hour on ice. Cells were also counterstained with Hoechst 33342 before the final wash and imaged.

Cells were imaged using a Bio-Rad MRC 1024 laser scanning confocal microscope equipped with Argon-Krypton laser and 60X, NA 1.4 objective lens. Fluorescence images of cells were recorded with sequential excitation and emission conditions using appropriate optics. The acquired images were processed for co-localization analysis using Image J1.34s software. For the analysis of endogenous Bid, Bim or Bax, samples were imaged using a LSM 510 Meta laser scanning confocal microscope (Carl Zeiss, Jena, Germany) equipped with Argon, Helium-Neon lasers and 63X, NA 1.4 objective. The fluorescence images for GFP tagged, Alexa Fluor 555 and Cy5 labeled proteins were acquired with sequential excitation (488 nm, 543 nm and 633 nm respectively) and emission conditions using appropriate optics. The nuclear morphology images were acquired using two photon excitation of single plane with 750 nm pulsed laser (Spectra-Physics, CA). Images were processed for co-localization analysis using Image J1.34s and LSM Meta (Carl Zeiss, Jena, Germany) software.

### Transgenic flies and genetic interaction studies

Bax-GFP, Bax 30–192-GFP and Bcl-2 were cloned in the UAS vector pUAST. The resulting plasmids were injected into *yw; +; Ki P(Δ2-3ry+) *embryos using standard procedures. Several independent transformant lines were obtained by P-element transformation for each transgene. A minimum of two lines in each case were characterized for further analysis. Bax-GFP insertion was mapped to chromosomes III and I for BA64III and BA13I transgenic lines respectively. Bax 30–192-GFP insertion was mapped to chromosome II and I for DBA8II and DBA8IA fly lines respectively. Bcl-2 insertion was mapped to chromosome I and III for BC57I and BC24III fly lines respectively. A single copy transformant fly line for each transgene was crossed to flies carrying *vestigial (vg)-GAL4*, *twist-GAL4*, *eyeless-GAL4*, *scalloped-GAL4 *or *collagen-GAL4*. Flies were raised on standard *Drosophila *medium at 25°C.

## Authors' contributions

NP contributed to the design and performance of experiments and the genetic analysis. CK contributed to the sequence analysis. VD carried out the immunoprecipitation analysis. LP performed functional assays with the site-directed mutant. RS coordinated and performed the sequence alignment and analysis, contributed to the design of experiments and the writing of the paper. AS contributed to the design and conducted experiments and the writing of the paper. All authors have read and approved the final manuscript.

## References

[B1] Martinou JC, Green DR (2001). Breaking the mitochondrial barrier. Nat Rev Mol Cell Biol.

[B2] Green DR, Kroemer G (2004). The pathophysiology of mitochondrial cell death. Science.

[B3] Adams JM, Cory S (1998). The Bcl-2 protein family: arbiters of cell survival. Science.

[B4] Kelekar A, Thompson CB (1998). Bcl-2-family proteins: the role of the BH3 domain in apoptosis. Trends Cell Biol.

[B5] Simonen M, Keller H, Heim J (1997). The BH3 domain of Bax is sufficient for interaction of Bax with itself and with other family members and it is required for induction of apoptosis. Eur J Biochem.

[B6] Simonian PL, Grillot DA, Andrews DW, Leber B, Nunez G (1996). Bax homodimerization is not required for Bax to accelerate chemotherapy-induced cell death. J Biol Chem.

[B7] Sattler M, Liang H, Nettesheim D, Meadows RP, Harlan JE, Eberstadt M, Yoon HS, Shuker SB, Chang BS, Minn AJ, Thompson CB, Fesik SW (1997). Structure of Bcl-x_L_-Bak peptide complex: recognition between regulators of apoptosis. Science.

[B8] Petros AM, Nettesheim DG, Wang Y, Olejniczak ET, Meadows RP, Mack J, Swift K, Matayoshi ED, Zhang H, Thompson CB, Fesik SW (2000). Rationale for Bcl-x_L_/Bad peptide complex formation from structure, mutagenesis, and biophysical studies. Protein Sci.

[B9] Liu X, Dai S, Zhu Y, Marrack P, Kappler JW (2003). The structure of a Bcl-X_L_/Bim fragment complex: Implications for Bim Function. Immunity.

[B10] Chen L, Willis SN, Wei A, Smith BJ, Fletcher JI, Hinds MG, Colman PM, Day CL, Adams JM, Huang DC (2005). Differential targeting of prosurvival Bcl-2 proteins by their BH3-only ligands allows complementary apoptotic function. Mol Cell.

[B11] Wolter KG, Hsu YT, Smith CL, Nechustan A, Xi XG, Youle RJ (1997). Movement of Bax from the cytosol to mitochondria during apoptosis. J Cell Biol.

[B12] Khaled AR, Kim K, Hofmeister R, Muegge K, Durum SK (1999). Withdrawal of IL-7 induces Bax translocation from cytosol to mitochondria through a rise in intracellular pH. Proc Natl Acad Sci USA.

[B13] Linseman DA, Butts BD, Precht TA, Phelps RA, Le SS, Laessig TA, Bouchard RJ, Florez-McClure ML, Heidenreich KA (2004). Glycogen synthase kinase-3beta phosphorylates Bax and promotes its mitochondrial localization during neuronal apoptosis. J Neurosci.

[B14] Kuwana T, Newmeyer DD (2003). Bcl-2-family proteins and the role of mitochondria in apoptosis. Curr Opin Cell Biol.

[B15] Cartron PF, Gallenne T, Bougras G, Gautier F, Manero F, Vusio P, Meflah K, Vallette FM, Juin P (2004). The first alpha helix of Bax plays a necessary role in its ligand-induced activation by the BH3-only proteins Bid and PUMA. Mol Cell.

[B16] Kuwana T, Bouchier-Hayes L, Chipuk JE, Bonzon C, Sullivan BA, Green DR, Newmeyer DD (2005). BH3 domains of BH3-only proteins differentially regulate Bax-mediated mitochondrial membrane permeabilization both directly and indirectly. Mol Cell.

[B17] Kim H, Rafiuddin-Shah M, Tu HC, Jeffers JR, Zambetti GP, Hsieh JJ, Cheng EH (2006). Hierarchical regulation of mitochondrion-dependent apoptosis by BCL-2 subfamilies. Nat Cell Biol.

[B18] Oh KJ, Barbuto S, Pitter K, Morash J, Walensky LD, Korsmeyer SJ (2006). A membrane-targeted BID BCL-2 homology 3 peptide is sufficient for high potency activation of BAX in vitro. J Biol Chem.

[B19] Wood DE, Thomas A, Devi LA, Berman Y, Beavis RC, Reed JC, Newcomb EW (1998). Bax cleavage is mediated by calpain during drug-induced apoptosis. Oncogene.

[B20] Yanase N, Takada E, Yoshihama I, Ikegami H, Mizuguchi J (1998). Participation of Bax-alpha in IFN-alpha-mediated apoptosis in Daudi B lymphoma cells. J Interferon Cytokine Res.

[B21] Cao X, Deng X, May WS (2003). Cleavage of Bax to p18 Bax accelerates stress-induced apoptosis, and a cathepsin-like protease may rapidly degrade p18 Bax. Blood.

[B22] Toyota H, Yanase N, Yoshimoto T, Moriyama M, Sudo T, Mizuguchi J (2003). Calpain-induced Bax-cleavage product is a more potent inducer of apoptotic cell death than wild-type Bax. Cancer Lett.

[B23] Gao G, Dou QP (2000). N-terminal cleavage of bax by calpain generates a potent proapoptotic 18-kDa fragment that promotes bcl-2-independent cytochrome C c release and apoptotic cell death. J Cell Biochem.

[B24] Jin KL, Graham SH, Mao XO, He X, Nagayama T, Simon RP, Greenberg DA (2001). Bax kappa, a novel Bax splice variant from ischemic rat brain lacking an ART domain, promotes neuronal cell death. J Neurochem.

[B25] Cartron PF, Oliver L, Martin S, Moreau C, LeCabellac MT, Jezequel P, Meflah K, Vallette FM (2002). The expression of a new variant of the pro-apoptotic molecule Bax, Baxpsi, is correlated with an increased survival of glioblastoma multiforme patients. Hum Mol Genet.

[B26] Nechustan A, Smith CL, Hsu YT, Youle RJ (1999). Conformation of the Bax C-terminus regulates subcellular location and cell death. EMBO J.

[B27] Goping IS, Gross A, Lavoie JN, Nguyen M, Jemmerson R, Roth K, Korsmeyer SJ, Shore GC (1998). Regulated targeting of BAX to mitochondria. J Cell Biol.

[B28] Cartron PF, Priault M, Oliver L, Meflah K, Manon S, Vallette FM (2003). The N-terminal end of Bax contains a mitochondrial-targeting signal. J Biol Chem.

[B29] Cartron PF, Arokium H, Oliver L, Meflah K, Manon S, Vallette FM (2005). Distinct domains control the addressing and the insertion of Bax into mitochondria. J Biol Chem.

[B30] Parikh N, Sade H, Kurian L, Sarin A (2004). The Bax N terminus is required for negative regulation by the mitogen-activated protein kinase kinase and Akt signaling pathways in T-cells. J Immunol.

[B31] Tusnady GE, Simon I (2001). The HMMTOP transmembrane topology prediction server. Bioinformatics.

[B32] Annis MG, Soucie EL, Dlugosz PJ, Cruz-Aguado JA, Penn LZ, Leber B, Andrews DW (2005). Bax forms multispanning monomers that oligomerize to permeabilize membranes during apoptosis. EMBO J.

[B33] Heimlich G, McKinnon AD, Bernardo K, Brdiczka D, Reed JC, Kain R, Kronke M, Jurgensmeier JM (2004). Bax-induced cytochrome c release from mitochondria depends on alpha-helices-5 and -6. Biochem J.

[B34] Kornbluth S, White K (2005). Apoptosis in Drosophila: neither fish nor fowl (nor man, nor worm). J Cell Sci.

[B35] Hsu YT, Youle RJ (1998). Bax in murine thymus is a soluble monomeric protein that displays differential detergent-induced conformations. J Biol Chem.

[B36] Adams JM, Cory S (2002). Apoptosomes: engines for caspase activation. Curr Opin Cell Biol.

[B37] Pan G, O'Rourke K, Dixit VM (1998). Caspase-9, Bcl-XL, and Apaf-1 form a ternary complex. J Biol Chem.

[B38] Song Q, Kuang Y, Dixit VM, Vincenz C (1999). Boo, a novel negative regulator of cell death interacts with Apaf-1. EMBO J.

[B39] Karbowski M, Lee YJ, Gaume B, Jeong SY, Frank S, Nechushtan A, Santel A, Fuller M, Smith CL, Youle RJ (2002). Spatial and temporal association of Bax with mitochondrial fission sites, Drp1, and Mfn2 during apoptosis. J Cell Biol.

[B40] Vinayagam A, Shi J, Pugalenthi G, Meenakshi B, Blundell TL, Sowdhamini R (2003). DDBASE2.0: updated domain database with improved identification of structural domains. Bioinformatics.

[B41] Johnson MS, Overington JP, Blundell TL (1993). Alignment and searching for common protein folds using a data bank of structural templates. J Mol Biol.

[B42] Zha H, Aime-Sempe C, Sato T, Reed JC (1996). Proapoptotic protein Bax heterodimerizes with Bcl-2 and homodimerizes with Bax via a novel domain (BH3) distinct from BH1 and BH2. J Biol Chem.

[B43] Zha H, Reed JC (1997). Heterodimerization-independent functions of cell death regulatory proteins Bax and Bcl-2 in yeast and mammalian cells. J Biol Chem.

[B44] Simonian PL, Grillot DA, Merino R, Nunez G (1996). Bax can antagonize Bcl-XL during etoposide and cisplatin-induced cell death independently of its heterodimerization with Bcl-XL. J Biol Chem.

[B45] Wang K, Gross A, Wakman G, Korsmeyer SJ (1998). Mutagenesis of the BH3 domain of BAX identifies residues critical for dimerization and killing. Mol Cell Biol.

[B46] Cartron PF, Oliver L, Juin P, Meflah K, Vallette FM (2004). The p18 truncated form of Bax behaves like a Bcl-2 homology domain 3-only protein. J Biol Chem.

[B47] Nouraini S, Six E, Matsuyama S, Krajewski S, Reed JC (2000). The putative pore-forming domain of Bax regulates mitochondrial localization and interaction with Bcl-X(L). Mol Cell Biol.

[B48] Willis SN, Fletcher JI, Kaufmann T, van Delft MF, Chen L, Czabotar PE, Ierino H, Lee EF, Fairlie WD, Bouillet P, Strasser A, Kluck RM, Adams JM, Huang DC (2007). Apoptosis initiated when BH3 ligands engage multiple Bcl-2 homologs, not Bax or Bak. Science.

[B49] Letai A, Bassik MC, Walensky LD, Sorcinelli MD, Weiler S, Korsmeyer SJ (2002). Distinct BH3 domains either sensitize or activate mitochondrial apoptosis, serving as prototype cancer therapeutics. Cancer Cell.

[B50] Willis SN, Chen L, Dewson G, Wei A, Naik E, Fletcher JI, Adams JM, Huang DC (2005). Proapoptotic Bak is sequestered by Mcl-1 and Bcl-xL, but not Bcl-2, until displaced by BH3-only proteins. Genes Dev.

[B51] Walensky LD, Pitter K, Morash J, Oh KJ, Barbuto S, Fisher J, Smith E, Verdine GL, Korsmeyer SJ (2006). A stapled BID BH3 helix directly binds and activates BAX. Mol Cell.

[B52] Zong WX, Li C, Hatzivassiliou G, Lindsten T, Yu QC, Yuan J, Thompson CB (2003). Bax and Bak can localize to the endoplasmic reticulum to initiate apoptosis. J Cell Biol.

